# Hepatitis A outbreak associated with consumption of dates, England and Wales, January 2021 to April 2021

**DOI:** 10.2807/1560-7917.ES.2021.26.20.2100432

**Published:** 2021-05-20

**Authors:** Tatiana Garcia Vilaplana, David Leeman, Koye Balogun, Siew Lin Ngui, Emily Phipps, Wazirzada M Khan, Incident Team, Sooria Balasegaram, Incident Team, Incident Team, Arwen Burns, Ben Sims, Charles Irish, Claire Bradshaw, Djamel Hamadache, Eleanor Blakey, James McCreadie, Jay Gordon, Joanne Freedman, Julia Heywood, Karthik Paranthaman, Lisa Kenney, Lucinda Slater, Maha O Saeed, Miranda Mindlin, Mona Dave, Nalini Iyanger, Neil MacDonald, Peter Lamb, Polly Ashmore, Rachel Pudney, Rebecca Greenwood, Rebecca Hams, Robert Smith, Samreen Ijaz, Sarah White, Sophie Nash, Amélie Kergozien, Anthony J Wilson, Carol Stocker, Caroline Handford, Sriram Balasingam, Tina Potter.

**Affiliations:** 1 These authors contributed equally to this article and share first authorship; 2 Immunisations and Countermeasures Division, National Infection Service, Public Health England, London, United Kingdom; 3 Field Epidemiology Training Programme, National Infection Service, Public Health England, London, United Kingdom; 4 Blood Safety, Hepatitis, STI and HIV Department, National Infection Service, Public Health England, London, United Kingdom; 5 Blood Borne Virus Unit, Virus Reference Department, National Infection Service, Public Health England, London, United Kingdom; 6 South London Health Protection Team, Public Health England, London, United Kingdom; 7 Public Health England and Food Standards Agency, London, United Kingdom; 8 South East and London Field Service, National Infection Service, Public Health England, London, United Kingdom

**Keywords:** hepatitis A, outbreak investigation, HAV, foodborne

## Abstract

We report a national hepatitis A virus (HAV) outbreak linked to the consumption of Medjool dates. Twenty-nine cases of three genetically related sequences have been identified. Epidemiological investigations identified a suspected product (adjusted odds ratio: 47.36; 95% confidence interval: 1.79–1,256.07; p = 0.021). Microbiological testing has confirmed the presence of HAV on dates recovered from two cases and the product has been recalled. Date consumption is currently likely to be increased in connection with Ramadan, with potential ongoing contamination risk.

An outbreak of genetically related hepatitis A virus (HAV) infections among people with no travel history was identified by the Public Health England (PHE) Virus Reference Department (VRD) in conjunction with local teams noting that the cases had eaten dates.

We describe investigations including case characteristics, phylogenetics, analytical studies, and control measures. We aim to flag the possible risk of hepatitis A to populations in other countries through the consumption of contaminated dates, particularly as Ramadan, which is associated with an increase in consumption of dates, began on 12 April 2021, and hepatitis A has a long incubation period of 15 to 50 days.

## Case definition

A confirmed case was defined as a laboratory-confirmed HAV infection with one of three clustered sequences (sequences VRD21_HAV005, VRD21_HAV009 and VRD21_HAV020) and onset date from 1 January 2021 in England or Wales, no travel history or contact with a suspected or confirmed HAV case in the 60 days before onset. A probable case was a laboratory-confirmed HAV infection, with no or pending sequencing result, and with an epidemiological link to a confirmed HAV case with one of the three clustered sequences.

## Outbreak description 

**Figure 1:**
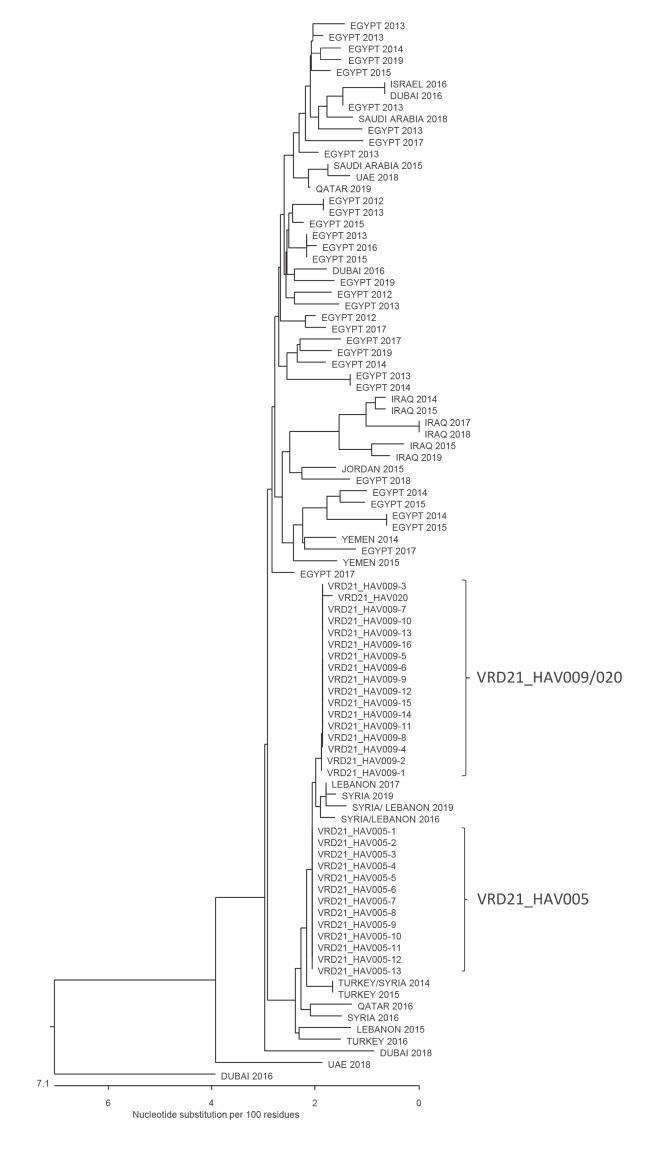
Phylogenetic tree of hepatitis A virus outbreak sequences associated with date consumption (n = 30), in a background of Middle Eastern sequences generated in the Public Health England Virus Reference Department, England and Wales, January–April 2021

Samples from all locally diagnosed HAV infections in England and Wales are routinely sent to the VRD for characterisation. The outbreak cases had HAV from three closely related Middle Eastern genotype IB sequences (≤ 2 bp different in a 505 bp segment) which clustered most closely with those found in travellers returning from Syria and Lebanon (Figure 1). The sequences have been submitted to the European Nucleotide Archive (ENA), accession numbers OD998295–OD998297. 

**Figure 2:**
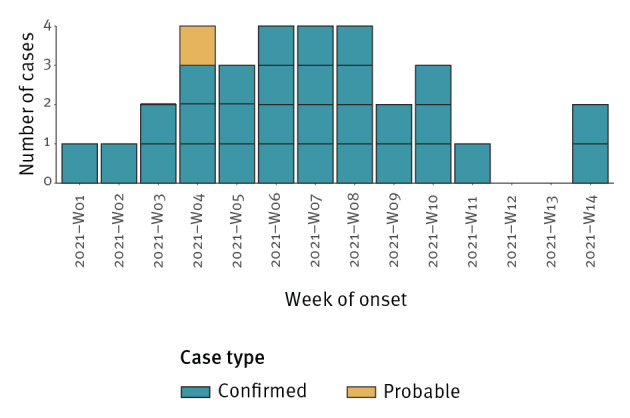
Confirmed and probable cases of hepatitis A virus infection associated with date consumption, by week of onset of symptoms, England and Wales, January–April 2021 (n = 31)

By 18 May, there have been 31 cases (30 confirmed and one probable). Cases had a median age of 60 years (range: 6–93), were predominately white British (21/31) and were geographically dispersed in England and Wales with symptom onset from 1 January 2021 (Figure 2). There was a slightly higher proportion of female cases (17/31). More than three quarters of cases were hospitalised (25/31) (Table 1).

Two cases had unclear date of first symptoms and are plotted using date of jaundice.

**Table 1:** Case characteristics of confirmed and probable cases of hepatitis A virus infection associated with date consumption, England and Wales, January–April 2021 (n = 31)

Characteristic	n	%
Sex		
Female	17	55
Male	14	45
Age (years)		
< 18	3	10
18–29	3	10
30–39	3	10
40–49	1	3
50–59	4	13
60–69	10	32
70–84	6	19
≥ 85	1	3
Median age (range)	60 (6–93)	
Ethnicity		
White (British)	21	68
White (other)	5	16
Asian (other)	1	3
Unknown	4	13
Hospital admission		
Admitted	25	81
Not admitted	5	16
Unknown	1	3
Jaundice		
Jaundiced	29	94
Not jaundiced	2	6
Unknown	0	0

## Analytical studies

Two case–case studies were undertaken to investigate the potential vehicle of transmission. Case data for both studies was extracted on 25 March 2021 (n = 21). 

Case-controls in Study 1 were not travel-associated, non-secondary HAV cases from the same period but with other sequences (n = 23). Enhanced surveillance questionnaires are completed with all HAV cases in England, including a food history if no recent travel is reported. We collected further product information (product name, description of packaging, batch numbers if available) from cases and case-controls reporting consumption of dates. Case-controls in Study 2 were cases from a *Salmonella* outbreak in England in autumn 2019 (n = 43), similar in age and sex. These cases had completed a full food history with similar food categories. Salmonellosis cases reported foods consumed in the week before onset of symptoms, whereas for HAV cases, it was 8 weeks, but questionnaires were administered several weeks after illness for both, thus having similar recall issues.

We conducted univariable analysis with attack rates and odds ratios with associated 95% confidence intervals (CI) and p values calculated for each food exposure using Pearson’s chi-squared or Fisher’s exact test. Variables with a p value < 0.1 were included in a multivariable model using a forward step approach. A Firth regression model was used because of total separation in some variables.

Case-controls were broadly comparable to cases for Study 1 (median age: 40 years, range 4–72, 13/23 female) and Study 2 (median age: 60 years, range 0–98, 28/43 female) and there was no statistical evidence of an association with sex or age in either study.

Results of both studies indicated an association with consumption of dates (Table 2). In Study 1, stratified by date product, Product A, a Medjool date product consumed by 11 of 29 cases, was strongly associated with being a case (adjusted odds ratio (aOR): 47.36; 95% CI: 1.79–1,256.07; p = 0.021).

**Table 2:** Results of multivariable analysis for two case–case studies, hepatitis A outbreak associated with consumption of fresh dates, England and Wales, as on 25 March 2021

Study 1: Hepatitis A cases with non-outbreak sequences as case-controls							
**Exposure**	**Cases** **(n = 21)**		**Controls** **(n = 23)**		**aOR**	**95% CI**	**p value**
	**n**	**%**	**n**	**%**			
Dates							
Product A	11	52	1	4	47.36	1.79–1,256.07	0.021
Only other dates	7	33	5	22	2.65	0.43–46.41	0.296
Missing information	0	0	3	13	0.37	0.01–16.10	0.607
Other variables							
Salmon	12	57	1	4	7.16	0.96–53.15	0.054
Lettuce	16	76	8	35	10.49	0.47–236.14	0.139
**Study 2: *Salmonella* outbreak cases as case-controls**							
**Exposure**	**Cases** **(n = 21)**		**Controls** **(n = 43)**		**aOR**	**95% CI**	**p value**
	**n**	**%**	**n**	**%**			
Dates	18	86	4	9	58.87	4.72–734.51	0.002
Salmon	12	57	12	28	1.66	0.03–79.41	0.796
Herbs	16	76	12	28	11.68	1.00–136.65	0.05
Shellfish	11	52	7	16	1.61	0.03–94.62	0.819
Cucumber	13	62	24	56	6.15	0.39–96.81	0.197

aOR: adjusted odds ratio; CI: confidence interval.

## Food investigations

Food chain traceback undertaken by the Food Standards Agency (FSA) identified two batch numbers and one common product, Product A, Medjool dates imported from Jordan linked to one retailer in the United Kingdom (UK), from which 25 of 31 cases reported purchasing dates. This retailer had recently received stock from Jordan from a new supplier. 

Dates from three cases were submitted for bacterial and viral contamination testing. Results reported on 26 April 2021 showed two separate date packages from two different cases were positive for HAV. Both positive products were of Product A; one package was the only dates reported to be consumed by one case, and the second package was unopened and had been purchased in bulk at the same time as other dates that were consumed by that case. No extract from either positive product were available for sequencing.

Three cases did not consume dates and three reported only consuming dates purchased at other retailers, however for two cases, the dates consumed traced back to the same producer of Product A via a different supplier who has now suspended supply. There were no other epidemiological links between cases.

Possible links between the producer and other suppliers and retailers are being investigated by the FSA through the UK supply chain and in collaboration with the Jordanian authorities.

## Control measures

On identification of the outbreak, we established an incident management team (IMT) on 19 March with colleagues from the FSA. Discussions identified that washing the fresh dates would not be sufficient to remove any HAV contamination. On the basis of the information available, the retailer took the decision to voluntarily withdraw the product from sale in stores on 31 March 2021. After receiving the results of the epidemiological investigation, the retailer undertook a product recall on 13 April 2021. This recall was published in stores and online by the retailer and the FSA [1].

A frequently asked questions document was provided to local health protection teams and primary care providers in response to concern from members of the public who had consumed Product A.

A total of five secondary cases and 64 contacts of confirmed or probable cases were identified, of whom 10 were older than 60 years. As per standard national guidance, post-exposure prophylaxis in the form of HAV vaccination was arranged for close contacts of cases with no history of HAV vaccination or immunity and the administration of human normal immunoglobulin for those in high risk groups such as those aged over 60 years or with chronic liver disease [2]. 

Information on this UK incident was disseminated internationally via the International Health Regulations focal point on 29 March 2021 and via the International Food Safety Authorities Network (INFOSAN) emergency contact points (ECP’s) on 15 April 2021. At 18 May, there have not been any reports of similar outbreaks elsewhere.

## Ethical statement

PHE has legal permission, provided by Regulation 3 of The Health Service (Control of Patient Information) Regulations 2002, to process patient confidential information for national surveillance of communicable diseases and as such, individual patient consent is not required.

## Discussion

We report an ongoing national outbreak of HAV infections in England and Wales associated with consumption of Medjool dates imported from Jordan. Epidemiological analysis was conducted rapidly using a case–case method to allow control measures at the approaching start of Ramadan on 12 April 2021. Dates are a popular food for breaking fast during Ramadan and sales of dates increase considerably during this period. Consequently, there was a concern that the public health burden of this outbreak could escalate if there was a contaminated food product continuing to be sold.

HAV is not endemic in the UK and is mostly related to recent travel to countries with higher rates of hepatitis A. European countries are also considered to be non-endemic or have low endemicity [3]. A number of outbreaks including multi-country outbreaks associated with imported food products have been reported in the last decade, including examples involving pomegranate arils [4], dried tomatoes [5-7], berries [8,9] and dates [10].

The complexity of food supply chains has made it difficult to identify the potential source particularly as most retailers use multiple suppliers. While the recalled product is related to a large proportion of cases, there are cases who reported consuming dates only from other retailers. Food chain and product investigations are ongoing and potential links between growers, packers and suppliers explored. This poses a risk that contaminated products from the same grower may still be supplied to other retailers in the UK and internationally, although no other outbreaks have been reported yet. As the contaminated product has come from a grower in Jordan, collaboration is ongoing with Jordanian authorities.

The use of case–case studies allowed rapid identification of the source without recruitment of controls, however limitations include the risk that hepatitis A controls are more like cases than the population and a small sample size. We increased confidence in dates as the likely source through Study 2 with a different case–control group, from a different time period but still thought to be sufficiently comparable.

## Conclusion

We describe an outbreak of hepatitis A initially identified by local health protection teams and the Virus Reference Department and characterised by sequencing. Rapid epidemiological analysis identified the source as imported dates and provided evidence for implementing public health action. Microbiological analysis of the dates confirmed contamination.
